# Critical Assessment of the Anti-Inflammatory Potential of Usnic Acid and Its Derivatives—A Review

**DOI:** 10.3390/life13041046

**Published:** 2023-04-19

**Authors:** Wojciech Paździora, Irma Podolak, Marta Grudzińska, Paweł Paśko, Karolina Grabowska, Agnieszka Galanty

**Affiliations:** 1 Department of Pharmacognosy, Jagiellonian University Medical College, Medyczna 9, 30-688 Kraków, Poland; wpazdziora@interia.pl (W.P.);; 2 Department of Food Chemistry and Nutrition, Jagiellonian University Medical College, Medyczna 9, 30-688 Kraków, Poland

**Keywords:** usnic acid, anti-inflammatory, enantioselective

## Abstract

Inflammation is a response of the organism to an external factor that disrupts its natural homeostasis, and it helps to eliminate the cause of tissue injury. However, sometimes the body’s response is highly inadequate and the inflammation may become chronic. Thus, the search for novel anti-inflammatory agents is still needed. One of the groups of natural compounds that attract interest in this context is lichen metabolites, with usnic acid (UA) as the most promising candidate. The compound reveals a broad spectrum of pharmacological properties, among which anti-inflammatory properties have been studied both in vitro and in vivo. The aim of this review was to gather and critically evaluate the results of the so-far published data on the anti-inflammatory properties of UA. Despite some limitations and shortcomings of the studies included in this review, it can be concluded that UA has interesting anti-inflammatory potential. Further research should be directed at the (i) elucidation of the molecular mechanism of UA; (ii) verification of its safety; (iii) comparison of the efficacy and toxicity of UA enantiomers; (iv) design of UA derivatives with improved physicochemical properties and pharmacological activity; and (v) use of certain forms or delivery carriers of UA, especially in its topical application.

## 1. Introduction

Inflammation is a dynamic response of the organism to an external factor that disrupts its natural homeostasis—most commonly apathogenic microorganisms or physical agents. Generally, this process helps to eliminate the cause of tissue injury, but in some diseases, the body’s response is highly inadequate. The unfolding chronic inflammation can result in cellular destruction and damage to tissues, or even promote the development of some serious diseases, such as cancer [1]. The visual signs of inflammation predominantly include local redness and swelling, but also pain, heat, and loss of function [2]. These result from a response of the organism to inflammation agents that initiates the sequential process, which comprises the activation of phospholipase A2 followed by the release of arachidonic acid and a number of inflammatory mediators (e.g., proinflammatory TNF-α or IL-1, anti-inflammatory IL-10 or IL-13) [3,4]. These mediators are one of the possible important targets in the search for novel anti-inflammatory drugs.

**Figure 1:**
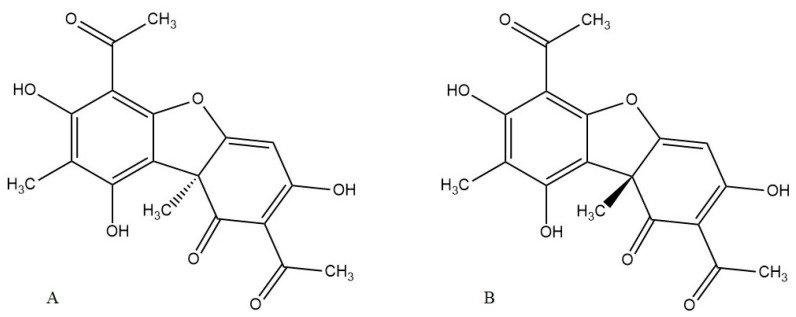
Structures of (−)-usnic acid (**A**) and (+)-usnic acid (**B**).

Nature is an almost inexhaustible source of bioactive compounds that can be considered new drug candidates for the treatment of various disorders, including inflammatory diseases. The classic anti-inflammatory drug used worldwide, aspirin, is derived from salicylic acid, a natural phenolic abundant in *Salix* sp. One of the groups of natural compounds that attract interest in this context is lichen metabolites. Lichens are composite organisms, that are primarily formed by the symbiotic co-existence of algal and/or cyanobacterial units and fungi, with the participation of basidiomycete yeasts and some bacterial communities [5]. A unique feature of lichens is that their metabolism stops under anhydrous conditions and returns to full metabolic activity under more favorable conditions. Despite their not very advanced evolutionary development, they contain primary and secondary metabolites, often with unique structures. One of the most interesting and promising, in terms of the pharmacological potential of lichen secondary metabolites, is usnic acid (2,6-diacetyl-7,9-dihydroxy-8,9b-dimethyl-1,3(2H,9bH)-dibenzofurandione), which is found at a particularly high content (up to 10%) in genera such as *Usnea*, *Alectoria*, *Cladonia*, *Lecanora*, *Ramalina*, and *Flavocetraria*. Usnic acid (UA) was first isolated in 1844 and, since then, its biological properties have been intensively studied, focusing mainly on antimicrobial, cytotoxic, antioxidant, and anti-inflammatory activities. It should be mentioned that this is a chiral compound (Figure 1). Even though there are many examples, among both synthetic and natural compounds, indicating that chirality can determine the activity observed, the enantioselectivity of usnic acid is still an open question, mainly due to scarce research data having been published so far [5].

None of the recent reviews on the pharmacological activity of usnic acid have specifically focused on its anti-inflammatory potential. Therefore, the present paper summarizes studies published to date on the anti-inflammatory properties of usnic acid in various in vitro and in vivo models and critically assesses the prospects of this compound with the view of using it as a lead structure for further chemical modifications. Furthermore, the enantioselectivity of the action of the compound is also discussed.

## 2. Materials and Methods

**Figure 2:**
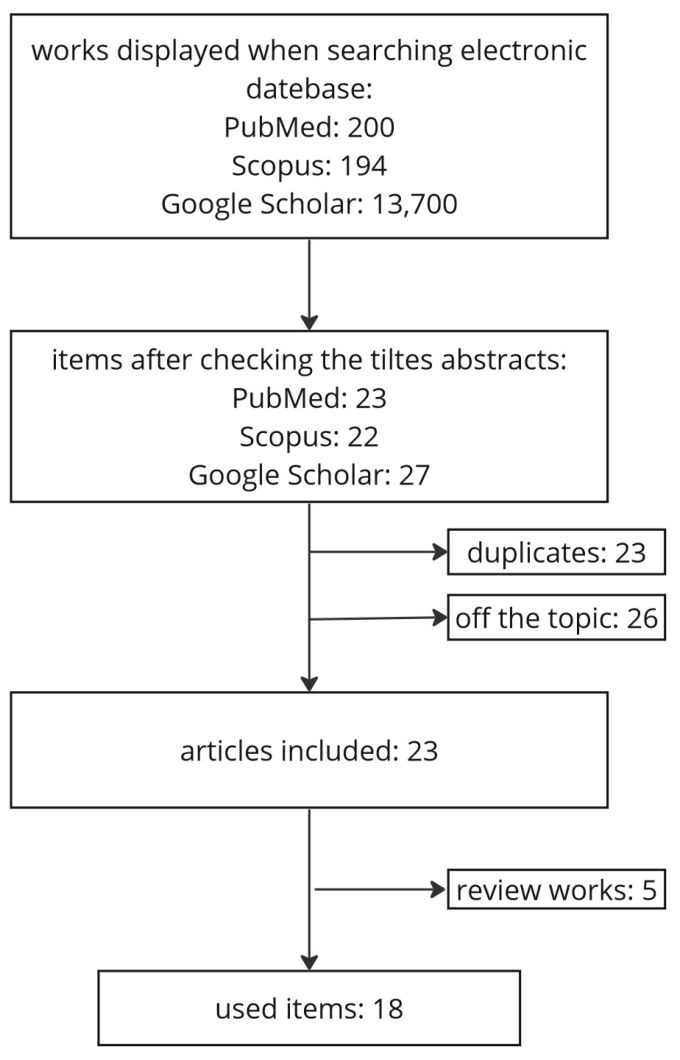
Searching strategy flowchart.

This review was conducted in accordance with the Preferred Reporting Items for Systematic Reviews and Meta-analyses (PRISMA). A literature search was conducted in the PubMed, Google Scholar, and Scopus databases, covering reports up to December 2022. Initially, the search term “usnic acid” was used, but it was too general and gathered papers on all of the different activities of this compound. For example, Scopus found 1313 articles containing this keyword. Accordingly, the following search terms were refined: “usnic acid anti-inflammatory”, “usnic acid antiinflammatory”, “usnic acid inflammation”, and “anti-inflammatory effects of usnic acid”. An additional criterion was the English language of the articles. After checking the titles and abstracts of the papers, 72 articles were selected. Then, after a deeper analysis of the full text, 23 duplicates and 26 studies were excluded. Further reports were found by checking the reference lists of previously identified scientific publications. Of the remaining 23 papers, 5 review articles were excluded, resulting in a total of 18 original studies used to prepare this review. The flow chart of the search method is shown in Figure 2.

## 3. Anti-Inflammatory Potential of Usnic Acid

### 3.1. Results from the In Vitro Studies

Several in vitro studies described the anti-inflammatory activity of usnic acid in an attempt to discover the potential mechanism at the cellular level. The published studies involved experiments on leukocytes or platelets isolated from blood, referring to the production of an eicosanoid inflammatory mediator, but also on RAW 264.7 macrophages stimulated by LPS, where NO or a different cytokine release was measured. Details of the experiments published to date and their results are shown in Table 1.

Kumar and Müller investigated the effect of (+)-UA on leukotriene B4 (LTB4) synthesis from bovine polymorphonuclear leukocytes. The compound was shown to have only a weak inhibitory effect on LTB4 biosynthesis, with an IC_50_ value of 42 ± 2.2 μM, whereas the values for the reference substances were 0.4 ± 0.21 μM (nordihydroguaiaretic acid) and 37 ± 4.6 μM (anthralin) [6]. The in vitro effect of (+)-UA on human plate-type 12(S)-lipoxygenase activity was also verified. However, in the concentration range of up to 100 μg/mL, UA did not inhibit the activity of the enzyme tested [7].

In the study on LPS-stimulated RAW 264.7 macrophages, significant reductions in the TNF-α level and NO production were observed after UA treatment at doses of 0.5–400 µM, with IC_50_ values of 12.8 µM and 4.7 µM, respectively. TNF-α mRNA expression was also inhibited. Western blot assay showed that UA suppressed LPS-induced inducible nitric oxide synthase (iNOS) protein synthesis and NF-κB p65 nuclear translocation in the cells tested. The degradation of I-κBα, a protein that inhibits NF-κB by masking the nuclear localization signals of NF-κB proteins and keeping them sequestered in an inactive state in the cytoplasm, was inhibited [8].

In a study by Huang et al. (2014), in the same cellular model, a similar decrease in the production of pro-inflammatory factors TNF-α, IL-1β, IL-6, and NO after treatment with UA at the concentrations of 1.5 and 10 µg/mL was noted. The observed decrease in the expression of TNF-α mRNA, COX-2 mRNA, and iNOS mRNA confirmed the activity of the compound at the cellular transcriptional and translational levels. At the same time, the mRNA levels of anti-inflammatory IL-10 and anti-inflammatory mediator heme oxygenase-1 (HO-1) increased significantly. Furthermore, a reduction in NF-κB activation was observed. These results indicated a dual effect of UA in reducing inflammation by stimulating the secretion of anti-inflammatory factors and inhibiting pro-inflammatory factors [9].

In our own studies on LPS-stimulated RAW 264.7 cells, the effects of both UA enantiomers at concentrations of 10 and 25 µg/mL were compared. A significant reduction in NO production was found for both concentrations, irrespective of the enantiomer used. In the case of IL-6, only the 25 µg/mL dose of both enantiomers had a significant effect on its release, whereas TNF-α production decreased only slightly, with no significant differences compared to control cells treated with LPS. In addition, the effect of both enantiomers on the expression of pro-inflammatory proteins: toll-like receptor 4 (TLR4), cytosolic phospholipase A2 (cPLA2), and cyclooxygenases (COX-1, COX-2) was also assessed. The inhibitory effect on TLR4 was observed at UA concentrations of 10 and 25 µg/mL, irrespective of the enantiomer used. Both UA enantiomers significantly and dose-dependently reduced cPLA2 synthesis in comparison to LPS-stimulated macrophages, with the strongest effect observed for (+)-UA at a concentration of 10 µg/mL. A dose-dependent decrease in COX-1 protein levels was observed for both enantiomers, but for (+)-UA only the higher dose made the effect significantly different from LPS-stimulated macrophages. Both UA enantiomers significantly decreased COX-2 protein levels, but for (−)-UA, the effect was dose-independent. Surprisingly, (+)-UA slightly increased COX-2 synthesis at the higher dose. The study showed a slight pro-inflammatory effect of (+)-UA, as the compound increased cPLA2 and COX-2 expression at the higher dose of 25 µg/mL, whereas no such effect was observed for (−)-UA [10].

A recent study tested the effect of UA on a broad panel of cytokines produced by unstimulated human breast cancer MCF-7 cells. In a concentration range of 0.62–15.64 µM, the compound significantly reduced the release of NO, vascular endothelial growth factor (VEGF), prostaglandin E2 (PGE2), cytokines (IL-2, CXCL 10, CXCL8, CCL2 (MCP-1), TNF-α, IL-6) in the cells, in a dose-dependent manner, compared to control cells. The compound also reduced the expression levels of COX-2 and iNOS genes [11].

### 3.2. Results from In Vivo Studies

It is noteworthy that UA was also tested in vivo in several models involving wound-healing, neurodegenerative, or lung diseases. Nevertheless, only one of these studies compared the impact of both enantiomers. Details of the experiments published to date and their results are shown in Table 2.

In probably the first published study, the anti-inflammatory potential of (+)-UA was evaluated in a rat model of induced chronic and acute inflammation; the compound’s activity was comparable to ibuprofen, which was used as a reference substance [12]. After a hiatus of almost a decade, studies exploring the anti-inflammatory potential of UA began to continue, targeting more specific problems, such as dermal inflammation and neurodegenerative- or lung disease-related inflammation.

Hard-to-heal wounds are a major health care problem. Inflammation is one of the natural stages of wound healing, forming an immune barrier against microbes. In many chronic wounds, there is clinically significant wound infection and/or excessive inflammation. The interesting efficacy of UA, in liposome form, in the treatment of burn wounds has been demonstrated in two experiments by the same research group (Table 2). In animals treated with UA, a significant improvement was observed in collagen quality and density [13], but also in granulation tissue and scar repair—better than in the case of the reference compound (sulfadiazine silver ointment) [14]. Despite some information on the allergic potential of UA [22], the authors did not observe such effects, even during the prolonged exposure time (up to 30 days). This could be explained by the use of a liposomal form in the study, which is safer for the body than the direct application of the compound [23].

Some recent studies, probably inspired by the lipophilic properties of UA and the proven ability of (−)-UA to cross the blood–brain barrier in vitro [24], have attempted to verify the exploitation of UA’s anti-inflammatory potential in neurodegenerative diseases. Cerebral ischemia causes oxidative stress, inflammation, and cell apoptosis due to oxygen deficiency. Astrocytes, some of the largest cells in the brain, are capable of producing pro-inflammatory factors under hypoxia, such as glial fibrillar acidic protein (GFAP). This protein is used as an indicator of astrocyte ischemia. Another indicator used to assess microglia activation is ionized calcium-binding adapter protein-1 (Iba-1). A study by Erfani et al. reported that UA significantly reduced the increase in caspase-3, GFAP, and Iba-1 values after cerebral ischemia in rats (Table 2). In addition, UA also revealed antioxidant activity, observed as an increase in superoxide dismutase (SOD) and glutathione synthetase (GSH) activity in hippocampal cells, which may support its anti-inflammatory activity against ischemia [16].

The neuroprotective effect of UA, resulting from its anti-inflammatory properties, was also suggested by the results of another study, with Parkinson’s-like brain changes induced by MPTP (1-methyl-4-phenyl-1,2,3,6-tetrahydropyridine) in mice (Table 2). UA suppressed motor dysfunction and effectively attenuated neurodegenerative changes (loss of dopaminergic neurons) in the substantia nigra and striatum. Moreover, the aforementioned markers, astrocytic GFAP and microglia Iba-1, were reduced in UA-treated animals, followed by the reduced activation of inducible NOS (an inflammation-related gene) in the substantia nigra. This confirms the ability of UA to inhibit inflammatory processes in the central nervous system [17].

One hypothesis for Alzheimer’s disease is that amyloid-β protein (Aβ) is deposited as amyloid fibers or non-fibrous amorphous aggregates in senile plaques, resulting in impaired neuronal transmission [25]. Aβ1−42 is one of the more cytotoxic amyloid isoforms, whose aggregation in the central nervous system causes neuroinflammation, oxidative stress, and apoptosis of the neuronal cells. Cazarin et al. tested different concentrations of UA enantiomers for the reduction in cognitive deficits, oxidative imbalance, and inflammation after the injection of Aβ1-42 into female mice (Table 2). This compound was chosen because, according to the authors, its structure reveals some similarities to galantamine, a drug used in Alzheimer’s disease. This was further supported by the results of their in silico experiment, where both UA enantiomers exhibited a complex-receptor interaction with acetylcholinesterase (AchE), similar to that of galantamine. In addition, both enantiomers revealed nootropic properties, observed as improved learning and memory in animals in various tests, and also reduced the activity of myeloperoxidase (MPO) and lipid hydroperoxides (LOOH) in the cortex and hippocampus and IL-1β levels in the hippocampus, without an effect on TNF-α. Despite the use of two enantiomers, the authors did not discuss differences in their activity [18].

The anti-inflammatory properties of UA have also been assessed for acute lung injury and acute respiratory distress syndrome, inflammatory diseases characterized by lung infiltrates, pulmonary edema, or hypoxemia, but also by a rapid overproduction of pro-inflammatory cytokines and chemokines, with a mortality rate of up to 40%. Both diseases are serious, with a long course for which there is still no effective treatment [26]. Zu-Qing Su et al. investigated the effect of UA on LPS-induced acute lung injury (ALI) in mice (Table 2). The application of UA significantly reduced mortality in mice with ALI, as well as neutrophils, macrophage levels, and the production of the studied cytokines in bronchoalveolar lavage fluid. However, the amount of anti-inflammatory IL-10 in the UA group was lower than in the LPS group. These results may be related to the suppressive effect of UA on neutrophil infiltration, which led to a reduction in the number of neutrophils in the lavage fluid. IL-10, as a counter-regulatory cytokine, is known to be produced more intensively after the increase in TNF-α production induced by LPS, which may also explain the high IL-10 content in the LPS group. Furthermore, the levels of myeloperoxidase (MOP), malondialdehyde (MDA), and H_2_O_2_ were significantly reduced, while the observed increase in SOD and GSH activities indicated the antioxidant properties of the compound [20]. In an experiment by Huang et al., lung fibrosis was induced with bleomycin in mice, and the impact of UA on selected markers, such as SOD, MDA, transforming growth factor beta 1 (TGF-β1), TNF-α, IL-1β, and IL-6, were investigated. Bleomycin caused a significant increase in MDA concentration and a decrease in SOD activity in the samples tested. The compound effectively inhibited MDA levels and reversed the bleomycin-induced decrease in SOD activity, and this effect was comparable to that of the reference prednisone acetate. In addition, there was a significant decrease in the expression of the cytokines tested, as recorded for UA, in a dose-dependent manner, thereby reducing inflammation in the lung tissue [21].

## 4. Anti-Inflammatory Potential of Synthetic Usnic Acid Derivatives In Vitro and In Vivo

Due to the documented anti-inflammatory effects of usnic acid, attempts have also been made to modify its structure in order to obtain synthetic derivatives, with improved physicochemical and anti-inflammatory properties. The studies were mainly conducted within in vitro models on LPS-stimulated cells of various origins, but two in vivo experiments have also been described. Details of the experiments published to date and their results are shown in Table 3.

**Figure 3:**
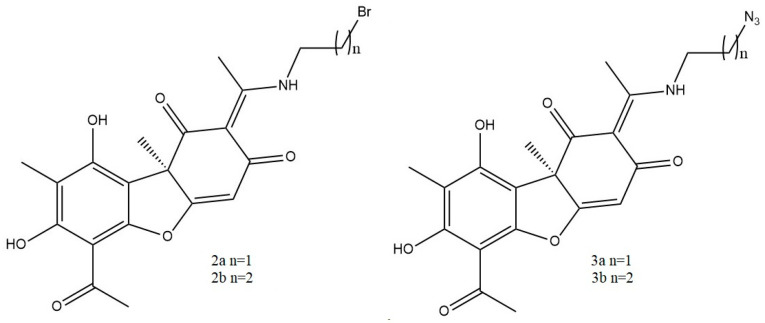
Intermediates of the target UA derivatives synthesis, according to Vanga et al. [27].

**Figure 4:**
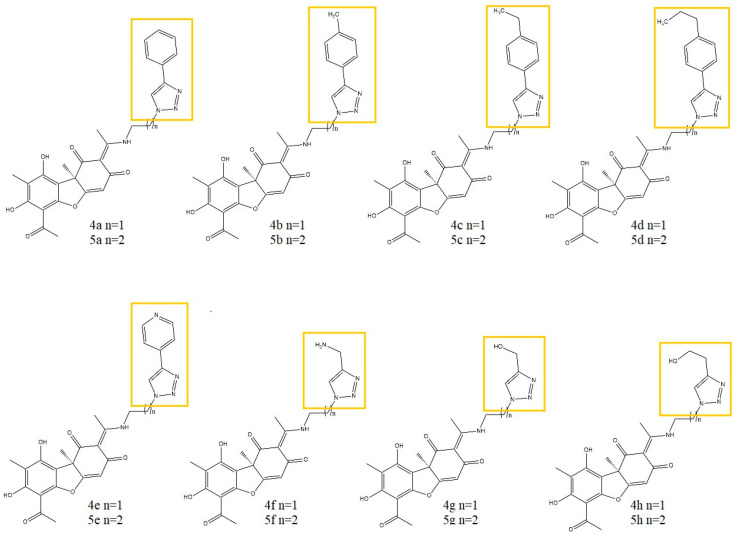
Structures of the synthesized UA triazole derivatives according to Vanga et al. [27], with the modification of the parent structure marked with yellow frames.

Vanga et al. [27], synthesized sixteen novel (+)-UA-based triazole hybrids and evaluated their in vitro anti-inflammatory potential against TNF-α and IL-1β release in the LPS-stimulated human lymphoma U937 cell line. Four intermediates (Figure 3) of the target synthesis (which were also included in the study) and sixteen synthesized triazole derivatives (Figure 4) showed promising anti-inflammatory activity against TNF-α, with IC_50_ values ranging from 1.40 to 5.70 μM compared to an IC_50_ > 100 μM for the parent compound.

The authors suggest that the triazole ring with an aliphatic side chain may be responsible for the increase in anti-inflammatory activity. Two of the triazole derivatives, 5f and 5h (Figure 4), were the most promising in terms of activity, as their IC_50_ (1.40 and 1.88 μM) were most similar to the values obtained for prednisolone (IC_50_ 0.52 μM), used as a reference drug. Interestingly, two of the intermediates tested (compounds 2a and 2b, Figure 3) showed stronger activity than some of the final triazole derivatives [27].

**Figure 5:**
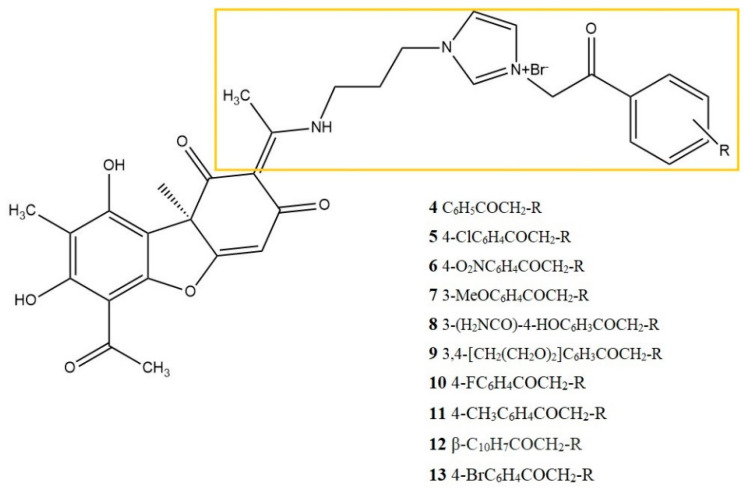
Structures of imidazolium salts of (+)-UA, according to Somasekhar et al. [28], with the modification of parent structure marked with yellow frames.

In a subsequent study, the same group of authors synthesized ten new (+)-UA imidazolium salts, which were evaluated for the in vitro anti-inflammatory potential of TNF-α and IL-1β on the LPS-stimulated human lymphoma U937 cell line. The three most active synthesized derivatives inhibited the release of TNF-α and IL-1β in 80.1 and 25.4% (compound No. 5, Figure 5); 17.3 and 90.4% (compound No. 6, Figure 5); and 4.7 and 85.5% (compound No. 13, Figure 5), respectively. The values for the reference substance, dexamethasone, were 81.4% and 80.5%, respectively. The IC_50_ values of the three most active compounds (No. 5, No. 6, No. 13) ranged from 5.3 μM to 7.5 μM and were many times lower compared to the parent UA (>100 μM), while for dexamethasone the IC_50_ values were 1.5 and 2.9 μM, respectively. The authors noted that the introduction of an enamine group at the C-2 position of (+)-UA, as present in the derivatives 5, 6, and 13, significantly increased the assessed anti-inflammatory activity, compared to the parent compound. Moreover, these most active derivatives were also characterized by the presence of electron-withdrawing groups in the phenacyl moiety, such as chloro, nitro, and bromo groups, while compounds bearing aromatic or heteroaromatic substituents (4 and 8–12) were significantly less active [28].

**Figure 6:**
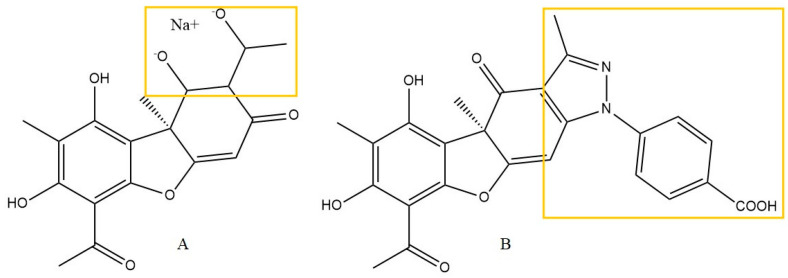
Structures of usnic acid sodium salt (**A**) and compound No 30 (**B**), according to Shi et al. [19], with the modification of parent structure marked with yellow frames.

Another study focused on the modification of the (+)-UA structure, retaining its anti-inflammatory properties, with additional properties to inhibit tau protein aggregation (an important element in the pathogenesis of Alzheimer’s disease). Twenty-five enamine derivatives and twenty-five hydrazines and hydrazides of (+)-UA were synthesized, but due to their better water solubility, the sodium salt of usnic acid (sodium usnate, SU, Figure 6A) was used as the reference parent compound. Compound No 30, with a substituted p-benzoic acid group (Figure 6B), appeared to be the most promising in terms of inhibition of tau aggregation.

This compound was also evaluated for the inhibition of LPS-induced nitric oxide release in the BV-2 mouse microglia cell line compared to SU. Interestingly, compound No 30 retained the anti-inflammatory effect of SUA and inhibited NO release by 41%, while it was significantly less toxic to the cells. The authors also assessed the neurotoxic and hepatotoxic potential of this derivative in vitro, and only minor effects on the viability of human neuroblastoma SH-SY5Y and LO2 hepatocytes were observed. Furthermore, in an in vivo Morris water maze test (see Table 2 for details), compound No 30 improved conventional reference spatial memory and cognitive abilities in okadaic acid-induced Alzheimer’s disease model rats [19].

Zhiheng Zhang et al. [15], investigated the sodium salt of usnic acid (SUA, Figure 6A), in the healing of an experimentally prepared wound in 8-week-old Wistar rats (see Table 2 for details). After 14 days of the experiment, a significant increase in wound healing activity was observed in the group treated with SUA (38.4 mg/kg), and the reference gentamicin sulfate (GA, 0.01%), compared to the untreated group. Furthermore, after the third day of treatment, the level of VEGF was significantly elevated in the SUA and GA groups, indicating faster skin regeneration processes. Unfortunately, UA alone was not included in the study; therefore, a comparison of the activity of SUA and the parent structure is not possible.

Despite the small number of experiments performed, the derivatives of UA designed so far, even as simple as its sodium salt, clearly demonstrate the utility of the compound’s parent structure to enhance its anti-inflammatory potential, both in vitro and in vivo.

## 5. Limitations of the Studies Included in the Review

Surprisingly few in vitro studies have been carried out so far, and their results still do not answer the question of UA’s anti-inflammatory mechanism. Only a general conclusion can be drawn, indicating an effect of the compound on the release and synthesis of inflammatory mediators, while more in-depth mechanistic studies are really needed. Although UA revealed significant anti-inflammatory activity in a relatively low concentration range of 5–25 µg/mL, the effect of dexamethasone used as the reference drug was observed at a much lower dose of 0.5 µg/mL. Furthermore, the control drug was used only in two studies, conducted on LPS-stimulated macrophages, and the results obtained in these studies are contradictory; the activity of UA was similar [9] or much weaker [10] than that of dexamethasone. The other two studies mentioned above did not include the reference drug [8,11].

Despite the interesting effects, the in vivo studies published so far can only be treated as preliminary observations, as a relatively small number of animals were used. The observed effects of usnic acid in reducing inflammation are particularly promising in wound-healing models using the liposomal form of UA, which may reduce the risk of UA’s allergic potential.

Although information on the pharmacokinetics of UA is limited, the experimental data suggest its high bioavailability [22], which may justify its potential oral use—for example, in neurodegenerative or lung diseases—as presented in the cited articles. However, in our opinion, the issue of UA toxicity, especially hepatotoxicity [29,30], was not taken into account during these experiments, as the cited studies generally lack information on the effects of UA on the liver or other organs. In one paper, the authors mentioned this problem [18], speculating that the effective UA dose of 25 mg/kg proven in their study was much lower than the toxic doses (<50 mg/kg) reported in some previous toxicological experiments [29]. As the effective dose of UA was 100 mg/kg in some studies included in this review, there is still a question about its safety.

The results obtained also cannot provide direct information on the superiority of one UA enantiomer over the other, as only two studies directly compared the activity of both enantiomers [10,18], while most of the other studies tested only (+)-. However, the significant differences in activity, as well as the small pro-inflammatory effect observed in our study for (+)-UA only, may suggest that this issue requires further research.

## 6. Conclusions

**Figure 7:**
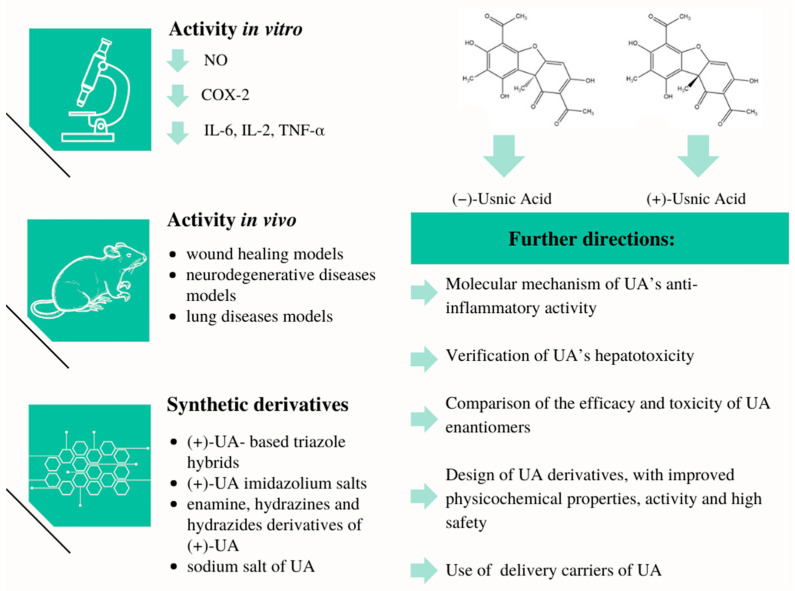
Summary of the anti-inflammatory effects of usnic acid and its derivatives.

Although the studies included in this review have some limitations and shortcomings, it can be concluded, without a doubt, that usnic acid has interesting anti-inflammatory potential. The summary of the results of the studies included in the review is presented in Figure 7.

Further research into its action in inflammatory diseases is highly anticipated, particularly directed at the (i) elucidation of the molecular mechanism of UA’s anti-inflammatory activity; (ii) verification of UA’s hepatotoxic properties, especially at the higher doses used; (iii) comparison of the efficacy and toxicity of UA enantiomers; (iv) design of UA derivatives, with improved physicochemical properties (especially solubility) and pharmacological activity, as well as high safety; and (v) use of certain forms or delivery carriers of UA, especially in its topical application.

## Figures and Tables

**Table 1:** Summary of in vitro anti-inflammatory activity of usnic acid.

In Vitro Model	Experimental Conditions	Effects	Ref.
bovine polymorphonuclear leukocytes (PMNL)	(+)-UA Reference: nordihydroguaiaretic acid, anthralin Groups: Ca-ionophore A23187-stimulated cells Methods: RP-HPLC (inhibition of LTB4 biosynthesis)	weak inhibitory effect on LTB4 biosynthesis IC_50_ 42 ± 2.2 μM for UA vs. 0.4 ± 0.21 μM for nordihydroguaiaretic acid vs. 37 ± 4.6 μM for anthralin	[6]
human platelets	(+)-UA (3.33–100 μg/mL) Reference: baicalein (IC_50_ = 24.6 μM) Methods: optical density, RP-HPLC (inhibition of platelet-type 12(S)-LOX)	no activity of UA up to 100 μg/mL	[7]
RAW 264.7 macrophages	UA (0.5–400 μM) Reference: none Groups: LPS-stimulated cells, untreated cells Methods: Griess reagent (NO), ELISA assay (TNF-α, iNOS, NF-κB, I-κB).	↓ TNF-α (dose-dependent effect)—IC_50_ 12.8 μM. ↓ NO (dose-dependent effect)—IC_50_ 4.7 μM. ↓ iNOS for 2.5, 5, 10 μM UA. ↓ NF-κB p65 for 2.5, 5, 10 μM UA. ↓ I-κB for 2.5, 5, 10 μM UA	[8]
RAW 264.7 cells	UA (1, 5, 10 μg/mL) Reference: dexamethasone 0.5 μg/mL Groups: LPS-stimulated cells, untreated cells (control) Methods: ELISA assay (TNF-α, IL-1β, IL-6, IL-10), Griess reagent (NO), RT-PCR (TNF-α mRNA, COX2 mRNA, iNOS mRNA, HO-1 mRNA), immunocytochemical assay (NF-κB), Western Blot (COX-2, HO-1)	dose-dependent effect—most effective dose 10 μg/mL UA. ↓ TNF-α, IL-1β, IL-6, NO, mRNA of TNF-α, mRNA of COX2, mRNA of iNOS, NF-κB. ↓ HO-1 mRNA (only 1 μg/mL)	[9]
RAW 264.7 cells	(+)-UA, (−)-UA (10, 25 µg/mL) Reference: dexamethasone 0.5 μg/mL Groups: LPS-stimulated cells, untreated cells (control) Methods: ELISA assay (TNF-α, IL-6) Griess reagent (NO), Western Blot (TLR4, cPLA2, COX-1, COX-2).	↓ NO for all variants ↓ IL-6 (only for (+)-UA 25 µg/mL) no influence on TNF-α production. ↓ TRL4 for all variants ↓ cPLA2 for all variant ↓ COX-1 for all variant ↑ COX-2 (only for (+)-UA 25 µg/mL)	[10]
MCF-7 breast cancer cells	UA (0.623–15,638 µM) Reference: none Groups: untreated (control) Methods: biochemical analysis (MDA, GSH), Griess reagent (NO), ELISA assay (PGE2, IL-2, IL-6, TNF-α), Bio-Plex assay (VEGF), RT-PCR (COX-2, iNOS)	dose-dependent effect—most effective dose 15,638 µM (group 6) UA ↓ NO, PGE2, IL-6, TNF-α, VEGF ↓ COX-2 and iNOS (by 81% in 6 groups compared to control) ↓ GSH (1,33-fold compared to control) ↑ MDA (1,62-fold compared to control)	[11]

UA, usnic acid; RP-HPLC, reverse-phase high-performance liquid chromatography; LTB4, leukotriene B4; TNF-α, tumor necrosis factor alpha; iNOS, inducible nitric oxide synthase; NF-κB, nuclear factor kappa B; I-κB, IκB kinase; LPS, lipopolysaccharide; IL-1β, interleukin-1β; IL-6, interleukin-6; IL-10, interleukin-10; NO, nitric oxide; RT-PCR, reverse transcription polymerase chain reaction; COX-2, cyclooxygenase-2; HO-1, heme oxygenase; TRL4, toll-like receptor 4; cPLA2, cytosolic phospholipase A2; COX-1, cyclooxygenase-1; MDA, malondialdehyde; GSH, glutathione; PGE2, prostaglandin E2; IL-2, interleukin-2; VEGF, vascular endothelial growth factor; ↓ decrease; ↑ increase.

**Table 2:** Summary on in vivo anti-inflammatory activity of usnic acid and its derivatives.

In Vivo Model	Experimental Conditions	Effects	Ref.
Induced chronic and acute inflammation in Wistar rats (n = 30)	(+)-UA: 25, 50, 100 mg/kg orally (p.o.) Reference: ibuprofen 100 mg/kg Different groups: untreated control Methods: volume of paw edema, weight of cotton pellets.	anti-edematous and anti-inflammatory effects of UA dose-dependent effect, with most effective dose of 100 mg/kg ↓ paw edema volume ↓ cotton pellet weight	[12]
**Wound healing models**			
Burn wound in male Wistar rats (n = 45)	Collagen film with liposomal UA: 330 mg/4 cm^2^, dermal application for 7, 14, and 21 days. Reference: no data Different groups: collagen film, collagen film with empty liposomes. Methods: histological assessment of inflammatory profile, epithelization rates, collagen deposition, mean of myofibroblasts for histological field.	day 7: moderate neutrophil infiltration over the entire wound surface (UA group) vs. infiltration only at the edges of the wound (other groups). day 14: ↓ inflammation with high plasma cell infiltration in UA group vs. others. day 21: slight inflammation in all groups. Content of highly undulating and dense type I and III collagen fibers, ↑ conversion of type III to type I collagen (UA group).	[13]
Burn wound in a porcine model (n = 9)	Gelatin-based membranes with liposomal UA: 127.02 mg/7 cm^2^, dermal application for 8, 18, and 30 days. Reference: ointment with silver sulfadiazine. Different groups: duoDerme^®^ dressing. Methods: histological assessment of burn healing grading, collagen deposition.	day 8: severe inflammation (UA group) vs. moderate (other groups). day 18: granulation tissue neoplasia advanced in all groups; more visible fibroblasts (UA group). day 30: 100% wound healing (UA and DuoDerme groups) vs. 80% (silver sulfadiazine ointment group).	[14]
Healing of wound in 8-week-old male Wistar rats (n = 64)	SUA: 38.4 mg/L in DMSO, daily dermal application for 21 days. Reference: gentamicin sulfate 0.01%. Different groups: untreated control, pure DMSO. Methods: wound area measured at 3, 7, 10, and 14 days after wounding. Histological assessment, immunohistochemistry analysis (VEGF).	↑ wound healing, re-epithelialization, ↓ inflammation (SUA and gentamicin groups). on day 21, full skin regeneration (SUA and gentamicin groups). VEGFT highest on day 1 (SUA, gentamicin) and day 3 (no treatment, pure DMSO). No significant differences between gentamicin and SU.	[15]
**Neurodegenerative diseases models**			
Model of cerebral ischemia/reperfusion by 20-min occlusion of the carotid arteries in male Wistar rats (n = 42)	UA: 25 mg/kg in DMSO, intraperitoneally (i.p.), 20 min of ischemia, and 48 h of reperfusion. Reference: no data Different groups: sham-operated, untreated control. Methods: Morris water maze task, spatial training test, spatial probe test, immunohistochemistry analysis (caspase-3, GFAP, Iba-1), biochemical assessment (SOD, GSH, MDA).	↑ caspase-3, GFAP, Iba-1 proteins ↑ SOD and GSH ↓ MDA	[16]
MPTP-induced Parkinson’s disease model in mice C57BL/6 (n = 40)	UA: 5 and 25 mg/kg intraperitoneally (i.p.) used daily for 10 days before MPTP-induced Parkinson’s disease. Reference: no data Different groups: sham control, untreated control. Methods: motor performance testing (rota-rod), immunocytochemical and immunochemical tests (Iba-1, GFAP, iNOS).	↓ astrocytic GFAP, microglial Iba-1, inducible nitric oxide synthase (iNOS) in the *substantia nigra* in UA group. dose-dependent effect—most effective dose 25 mg/kg UA	[17]
Aβ1-42-induced Alzheimer’s disease model in female mice (n = 81)	(R)-(+)- and (S)-(−)-UA: 25, 50, and 100 mg/kg, orally (p.o.) for 24 days. Reference: donepezil 2 mg/kg. Different groups: naïve, untreated control, sham-operated, Methods: open field test, novel object recognition test, Morris water maze task, Inhibitory-avoidance test, biochemical analysis (SOD, GSH, LOOH, MPO, IL-1β).	↑ SOD ((R)-(+)-UA (50 and 100 mg/kg), (S)-(−)-UA (100 mg/kg)) in the hippocampus. ↑ GSH ((R)-(+)-UA 100 mg/kg) in the hippocampus. ↓ LOOH, MPO (all variants) in the cerebral cortex and hippocampus. ↓ IL-1β only in the hippocampus (without (S)-(−)-UA 100 mg/kg). no effect on TNF-α.	[18]
Okadaic acid-induced memory impairment in male rats SD (n = 32)	UA derivative No 30 *: 5 and 10 mg/kg, intraperitoneally (i.p.) for 7 days after okadaic acid injection. Reference: no data Different groups: sham-operated, natrium chloratum 0.9%. Methods: Morris water maze task.	↑ memory and cognitive abilities of the derivative. dose-dependent effect—most effective dose 10 mg/kg UA. ↓ escape latency no impact on swimming speed.	[19]
**Lung diseases models**			
LPS-induced acute lung injury (ALI) in mice (n = no data)	UA: 25, 50, or 100 mg/kg used daily for 5 days intratracheal. Reference: dexamethasone 5 mg/kg. Different groups: naïve, untreated control. Methods: histological assessment of BALF, immunochemical analysis (MPO, MDA, TNF-α, IL-6, IL-10, IL-8, MIP-2, GSH, SOD).	dose-dependent effect—most effective dose 100 mg/kg UA ↓ mortality (50, 100 mg/kg) ↓ immune cells in the bronchoalveolar lavage fluid (BALF). ↓ MPO, MDA, TNF-α, IL-6, IL-10, IL-8, MIP-2 (50, 100 mg/kg). ↑ GSH and SOD (50, 100 mg/kg)	[20]
Bleomycin-induced lung fibrosis in mice (n = no data)	UA: 25, 50, or 100 mg/kg with bleomycin 15 mg/kg used daily for 21 days i.p. Reference: prednisone acetate 5 mg/kg. Different groups: natrium chloratum 0.9%, untreated control. Methods: histological assessments, immunochemical analysis (TGF-β1, TNF-α, IL-1β,IL-6, SOD, MDA).	dose-dependent effect—most effective dose 100 mg/kg UA. ↑ SOD, ↓ MDA (100 mg/kg UA) ↓ TGF-β1, TNF-α, IL-1β and IL-6 (all doses of UA).	[21]

UA, usnic acid; SUA, sodium usnic acid; DMSO, dimethyl sulfoxide; VEGF, vascular endothelial growth factor; GFAP, glial fibrillary acidic protein; Iba-1, ionized calcium-binding adapter molecule 1; SOD, superoxide dismutase; GSH, glutathione; MDA, malondialdehyde; MPTP, 1-methyl-4-phenyl-1,2,3,6-tetrahydropyridine; iNOS, inducible nitric oxide synthase; LOOH, lipid hydroperoxide; MPO, myeloperoxidase; IL-1β, interleukin-1β; TNF-α, tumor necrosis factor alpha; BALF, bronchoalveolar lavage fluid; IL-6, interleukin-6; IL-10, interleukin-10; IL-8, interleukine-8; MIP-2, macrophage inflammatory protein-2; TGF-β1, transforming growth factor β1, * UA derivative No 30, according to [19]; ↓ decrease; ↑ increase.

**Table 3:** Summary of in vitro anti-inflammatory activity of usnic acid derivatives.

Cellular Model	Experimental Conditions	Effects	Ref.
lymphoma U937 cells	16 derivatives of UA (10 μM) Reference: prednisolone 10 μM groups: LPS-stimulated cells Methods: ELISA assay (IL-1β, TNF-α)	Compounds No 5f and 5h—the highest scores ↓ TNF-α by 90.94% and 83.75%, respectively, vs. prednisolone at 60.69% ↓ IL-1β by 12.4% and 16.74%, respectively, vs. prednisolone at 46.11% IC_50_ 1. (No 5f) and 1.88 (No 5h) vs. prednisolone 0.52	[27]
lymphoma U937 cells	UA derivatives No 4-13 (10 μM) Reference: dexamethasone 10 μM. Different groups: LPS-stimulated cells, Methods: ELISA assay (IL-1β, TNF-α)	Compounds No 5, 6, and 13—the highest scores ↓ TNF-α by 80.1%, 17.3%, and 4.7%, respectively, vs. dexamethasone at 81.4% ↓ IL-1β by 25.4%, 90.4%, 85.4%, respectively, vs. dexamethasone at 80.5% IC_50_ from 5.3 ± 0.01 (No 5) to 7.5 ± 0.1 (No 6) vs. dexamethasone from 1.5 ± 0.04 to 2.9 ± 0.05	[28]
microglia BV2 cells	UA derivative No 30 (2.5, 5, 10 μM) Reference: sodium usnate 10 μM Different groups: LPS-stimulated cells Methods: Griess reagent (NO)	Dose-dependent effect—most effective dose 10 µM ↓ NO by 41% (10 μM)	[19]

UA, usnic acid; LPS, lipopolysaccharide; TNF-α, tumor necrosis factor alpha; IL-1β, interleukin-1β; NO, nitric oxide. ↓ decrease.

## Data Availability

Not applicable.

## References

[1] Marchi S., Guilbaud E., Tait S.W., Yamazaki T., Galluzzi L. (2023). Mitochondrial control of inflammation. Nat. Rev. Immunol..

[2] Nunes C.D.R., Barreto Arantes M., Menezes de Faria Pereira S., Leandro da Cruz L., de Souza Passos M., Pereira de Moraes L., Vieira I.J.C., Barros de Oliveira D. (2020). Plants as Sources of Anti-Inflammatory Agents. Molecules.

[3] de Cássia da Silveira e Sá R., Andrade L.N., de Sousa D.P. (2013). A review on anti-inflammatory activity of monoterpenes. Molecules.

[4] Karpel E. (2001). Mediatory ogólnoustrojowej odpowiedzi zapalnej—Znaczenie w praktyce klinicznej intensywnej terapii. Anestezjol. Intensywna Ter..

[5] Galanty A., Paśko P., Podolak I. (2019). Enantioselective activity of usnic acid: A comprehensive review and future perspectives. Phytochem. Rev..

[6] Kumar S., Muller K. (1999). Lichen Metabolites. 2. Antiproliferative and cytotoxic activity of gyrophoric, usnic, and diffractaic acid on human keratinocyte growth. J. Nat. Prod..

[7] Bucar F., Schneider I., Ogmundsdóttir H., Ingólfsdóttir K. (2004). Anti-proliferative lichen compounds with inhibitory activity on 12(S)-HETE production in human platelets. J. Phytomed..

[8] Jin J., Li C., He L. (2008). Down-regulatory effect of usnic acid on nuclear factor-κB-dependent tumor necrosis factor-α and inducible nitric oxide synthase expression in lipopolysaccharide-stimulated macrophages RAW 264.7. Phytother. Res..

[9] Huang Z., Tao J., Ruan J., Li C., Zheng G. (2014). Anti-inflammatory effects and mechanisms of usnic acid, a compound firstly isolated from lichen *Parmelia saxatilis*. J. Med. Plant Res..

[10] Galanty A., Zagrodzki P., Gdula-Argasińska J., Grabowska K., Koczurkiewicz-Adamczyk P., Wróbel-Biedrawa D., Podolak I., Pękala E., Paśko P. (2021). A comparative survey of anti-melanoma and anti-inflammatory potential of usnic acid enantiomers—A comprehensive in vitro approach. Pharmaceuticals.

[11] Yildirim M., Degirmenci U., Akkapulu M., Gungor M., Oztornacı R.O., Berkoz M., Comelekoglu U., Yalın A.E., Yalın S. (2022). Anti-inflammatory effects of usnic acid in breast cancer. Russ. J. Bioorg. Chem..

[12] Vijayakumar C.S., Viswanathan S., Reddy M.K., Parvathavarthini S., Kundu A.B., Sukumar E. (2000). Anti-inflammatory activity of (+)-usnic acid. Fitoterapia.

[13] Nunes P.S., Albuquerque-Júnior R.L.C., Cavalcante D.R.R., Dantas M.D.M., Cardoso J.C., Bezerra M.S., Souza J.C.C., Russo Serafini M., Quitans L.J., Bonjardim L.R. (2011). Collagen-based films containing liposome-loaded usnic acid as dressing for dermal burn healing. Biomed. Res. Int..

[14] Nunes P.S., Rabelo A.S., Campos de Souza J.C., Vasconcelos Santana B., Monteiro Menezes da Silva T., Russo Serafini M., Dos Passos Menezes P., Dos Santos Lima B., Cordeiro Cardoso J., Santana Alves J.C. (2016). Gelatin-based membrane containing usnic acid-loaded liposome improves dermal burn healing in a porcine model. Int. J. Pharm..

[15] Zhang Z., Zheng Y., Li Y., Bai H., Ma T., Song X., Zhao J., Gao L. (2018). The effects of sodium usnic acid by topical application on skin wound healing in rats. Biomed. Pharmacother..

[16] Erfani S., Valadbeigi T., Aboutaleb N., Karimi N., Moghimi A., Khaksari M. (2020). Usnic acid improves memory impairment after cerebral ischemia/reperfusion injuries by anti-neuroinflammatory, anti-oxidant, and anti-apoptotic properties. Iran. J. Basic Med. Sci..

[17] Lee S., Lee Y., Ha S., Chung H.Y., Kim H., Hur J.S., Lee J. (2020). Anti-inflammatory effects of usnic acid in an MPTP-induced mouse model of Parkinson’s disease. Brain Res. J..

[18] Cazarin C.A., Dalmagro A.P., Gonçalves A.E., Boeing T., Mota da Silva L., Correa R., Klein-Júnior L.C., Carlesso Pinto B., Savoldi Lorenzett T., Uchoa da Costa Sobrinho T. (2021). Usnic acid enantiomers restore cognitive deficits and neurochemical alterations induced by Aβ1-42 in mice. Behav. Brain Res..

[19] Shi C.J., Peng W., Zhao J.H., Yang H.J., Qu L.L., Wang C., Kong L.Y., Wang X.B. (2020). Usnic acid derivatives as tau-aggregation and neuroinflammation inhibitors. Eur. J. Med. Chem..

[20] Su Z.-Q., Mo Z.Z., Liao J.-B., Feng X.-X., Liang Y.-Z., Zhang X., Liu Y.-H., Chen X.-Y., Chen Z.-W., Su Z.-R. (2014). Usnic acid protects LPS-induced acute lung injury in mice through attenuating inflammatory responses and oxidative stress. Int. Immunopharmacol..

[21] Huang X.Q., Ai G.X., Zheng X.H., Liao H.J. (2019). Usnic acid ameliorates bleomycin-induced pulmonary fibrosis in mice via inhibition of inflammatory responses and oxidative stress. Trop. J. Pharm. Res..

[22] Wang H., Xuan M., Huang C., Wang C. (2022). Advances in research on bioactivity, toxicity, metabolism, and pharmacokinetics of usnic acid in vitro and in vivo. Molecules.

[23] da Silva Santos N.P., Nascimento S.C., Wanderley M.S.O., Pontes-Filho N.T., da Silva J.F., de Castro C.M.M.B., Santos-Magalhaes N.S. (2006). Nanoencapsulation of usnic acid: An attempt to improve antitumour activity and reduce hepatotoxicity. Eur. J. Pharm. Biopharm..

[24] Studzińska-Sroka E., Majchrzak-Celińska A., Zalewski P., Szwajgier D., Baranowska-Wójcik E., Kaproń B., Plech T., Żarowski M., Cielecka-Piontek J. (2021). Lichen-derived compounds and extracts as biologically active substances with anticancer and neuroprotective properties. Pharmaceuticals.

[25] Gaweł M., Potulska-Chromik A. (2015). Neurodegenerative diseases: Alzheimer’s and Parkinson’s disease. Postępy Nauk Med..

[26] Parekh D., Dancer R.C., Thickett D.R. (2011). Acute lung injury. Clin. Med..

[27] Vanga N.R., Kota A., Sistla R. (2017). Synthesis and anti-inflammatory activity of novel triazole hybrids of (+)-usnic acid, the major dibenzofuran metabolite of the lichen *Usnea longissima*. Mol. Divers..

[28] Somasekhar T., Javadi M., Sistla R. (2021). Synthesis of novel anti-inflammatory usnic acid-based imidazolium salts. Eur. Chem. Bull..

[29] Guo L., Shi Q., Fang J.L., Mei N., Ali A.A., Lewis S.M., Frankos V.H. (2008). Review of usnic acid and *Usnea barbata* toxicity. J. Environ. Health Part C.

[30] Croce N., Pitaro M., Gallo V., Antonini G. (2022). Toxicity of Usnic Acid: A Narrative Review. J. Toxicol..

